# A 56-year-old woman with breathlessness

**DOI:** 10.1136/heartjnl-2016-310611

**Published:** 2016-10-29

**Authors:** Alastair J Moss, Marc R Dweck, Peter D O'Kane

**Affiliations:** 1 Centre for Cardiovascular Science, University of Edinburgh, Royal Infirmary of Edinburgh, Edinburgh, UK; 2 Dorset Heart Centre, Royal Bournemouth Hospital, Bournemouth, UK

## Abstract

**Clinical introduction:**

A 56-year-old female with adult-onset asthma was admitted to the cardiology service with intermittent left-sided chest pain and progressive dyspnoea. Twelve months prior to this admission, she had received a course of prednisolone for bilateral anterior uveitis. Physical examination was unremarkable with blood sampling revealing a marked eosinophilia (eosinophil count 17.3×10^9^/L) and a perinuclear antineutrophil cytoplasmic antibody staining pattern on indirect immunofluorescence microscopy (myeloperoxidase antibodies 83 IU/mL). ECG demonstrated anterolateral T-wave inversion (see online supplementary figure S1). High-sensitivity troponin T was elevated at 100 ng/L. Invasive coronary angiography showed unobstructed coronary arteries. Echocardiography and cardiac magnetic resonance (CMR) were performed (figure 1).

**Question:**

What is the most appropriate therapy?

- Beta-blockade and ACE inhibition
- Methylprednisolone
- Intravenous antibiotics
- Endocardiectomy
- Implantable cardioverter defibrillator (ICD)

## Answer: B

The correct answer is methylprednisolone. Echocardiography demonstrated an apical mass with obliteration of the left ventricular apex and extensive apical late gadolinium enhancement confirmed on CMR. Left ventricular function was preserved with no evidence of thrombus formation (see online supplementary movies 1 and 2). In combination with the clinical presentation, these findings were suggestive of acute eosinophilic myocarditis with an apical eosinophilic infiltrate.^1^ Myeloperoxidase immunofluorescence pointed towards a unifying diagnosis of eosinophilic granulomatosis with polyangiitis (Churg-Strauss syndrome). This acute presentation differs from the ‘classical’ subacute appearance on CMR where there is usually ventricular thinning with superimposed endocardial thrombus. The first-line treatment is immunosuppression with methylprednisolone.^2^

**Figure 1:**
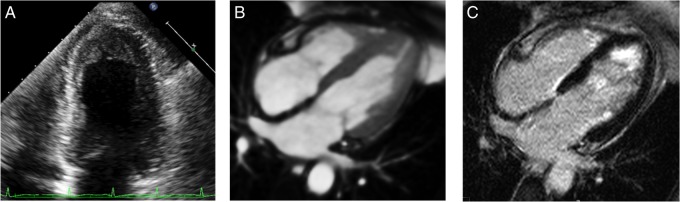
(A) Two-chamber echocardiogram. (B) Four-chamber steady-state free precession cardiac MRI. (C) Phase-sensitive inversion recovery cardiac MRI following gadolinium contrast.

10.1136/heartjnl-2016-310611.supp1 supplementary figure 12-lead electrocardiogram.

10.1136/heartjnl-2016-310611.supp2 supplementary movie Echocardiography video loop in modified four chamber view.

10.1136/heartjnl-2016-310611.supp3 supplementary movie Cine magnetic resonance sequence in four chamber view.

Hypereosinophilic syndrome with cardiac involvement has been characterised in three stages: an acute necrotic stage with eosinophilic infiltration, a subacute phase with thrombus formation overlying the disrupted endocardium and chronic fibrotic progression to a restrictive cardiomyopathy. In the acute stage, medical therapy for chronic systolic heart failure is not indicated in the context of preserved left ventricular function. Antibiotics are not indicated in the absence of bacterial infection. Endocardiectomy with preservation of the atrioventricular valve apparatus is advocated for advanced endomyocardial fibrosis to reduce ventricular filling pressures; however, it is not warranted during the acute phase.^3^ Primary ICD therapy is not indicated in the absence of recurrent ventricular tachyarrhythmias.^4^

The patient was treated with methylprednisolone and cyclophosphamide. After 12 months of treatment, she had fully recovered with resolution of her peripheral eosinophilia and cardiac findings. Eosinophilic myocarditis has a high mortality rate, but early identification and treatment can lead to a regression of fulminant myocardial necrosis.
