# Supplementary material for: A 56-year-old woman with breathlessness
Source: Heart. 2016 Oct 29;103(9):726. doi: 10.1136/heartjnl-2016-310611 (PMC5529977; doi:10.1136/heartjnl-2016-310611)
Supplement: supplementary figure — 12-lead electrocardiogram. [file heartjnl-2016-310611supp001.pdf]

SUPPLEMENTARY FIGURE 1: 12-lead electrocardiogram.

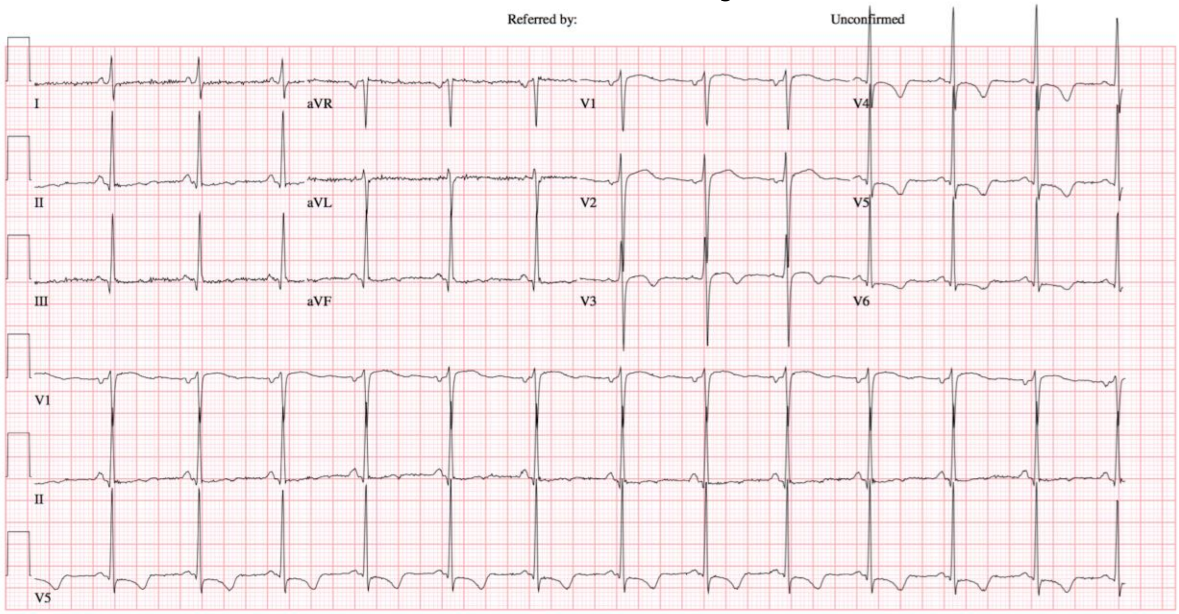

SUPPLEMENTARY MOVIE 1
Echocardiography video loop in modified four chamber view.

SUPPLEMENTARY MOVIE 2
Cine magnetic resonance sequence in four chamber view.
